# Cardiovascular responses to orthostasis and their association with falls in older adults

**DOI:** 10.1186/s12877-015-0168-z

**Published:** 2015-12-24

**Authors:** Brett H. Shaw, Thomas M. Loughin, Stephen N. Robinovitch, Victoria E. Claydon

**Affiliations:** Department of Biomedical Physiology and Kinesiology, Simon Fraser University, British Columbia, V5A 1S6 Canada; Department of Statistics and Actuarial Science, Simon Fraser University, British Columbia, V5A 1S6 Canada

**Keywords:** Orthostatic hypotension, Cerebrovascular hemodynamics, Older adults, Falling

## Abstract

**Background:**

Orthostatic hypotension (OH) refers to a marked decline in blood pressure when upright. OH has a high incidence and prevalence in older adults and represents a potential intrinsic risk factor for falls in these individuals. Previous studies have not included more recent definitions for blood pressure responses to orthostasis, including initial, delayed, and recovery blood pressure responses. Furthermore, there is little research examining the relationships between cerebrovascular functioning and falling risk. Therefore, we aimed to: (i) test the association between different blood pressure responses to orthostatic stress and retrospective falling history and; (ii) test the association between cerebrovascular responses to orthostatic stress and falling history.

**Methods:**

We tested 59 elderly residents in long term care facilities who underwent a passive seated orthostatic stress test. Beat-to-beat blood pressure and cerebral blood flow velocity (CBFV) responses were assessed throughout testing. Risk factors for falls and falling history were collected from facility records. Cardiovascular responses to orthostasis were compared between retrospective fallers (≥1 fall in the previous year) and non-fallers.

**Results:**

Retrospective fallers had larger delayed declines in systolic arterial pressure (SAP) compared to non-fallers (*p* = 0.015). Fallers also showed poorer early (2 min) and late (15 min) recovery of SAP. Fallers had a greater decline in systolic CBFV.

**Conclusions:**

Older adults with a positive falling history have impaired orthostatic control of blood pressure and CBFV. With better identification and understanding of orthostatic blood pressure impairments earlier intervention and management can be implemented, potentially reducing the associated risk of morbidity and mortality. Future studies should utilize the updated OH definitions using beat-to-beat technology, rather than conventional methods that may offer less accurate detection.

## Background

One third of individuals aged >65 years fall at least once each year [1]. In Canada, falls account for 85 % of injury-related hospitalizations [2]. Altered blood pressure responses, such as orthostatic hypotension (OH) have long been suggested to be a direct mechanism for an increased risk of falls in older adults through triggering spells of dizziness or loss of consciousness secondary to a transient decline in cerebral perfusion [3, 4]. Additionally, associations between OH and cognitive function or balance could also be indirectly linked to increased falling risk [3, 5]. A number of prospective longitudinal studies have attempted to assess the relationship between OH and falls; however, several thorough reviews of the literature have indicated that the available evidence does not clearly show that such a relationship exists [3, 6].

It is challenging to compare studies that examine OH, as there is great heterogeneity in methodological choices for the equipment and testing protocol used to measure OH, as well the definition used to identify the timing and magnitude of a blood pressure decline that constitutes OH [3, 6]. Most studies examining the relationship between OH and falls used single intermittent measurements of blood pressure to detect OH [3], and consequently did not consider important beat-to-beat blood pressure fluctuations that may contribute to OH [5]. Continuous beat-to-beat blood pressure measurements are reported to be more effective in detecting OH, and in discriminating blood pressure responses in fallers and non-fallers compared to intermittent manual sphygmomanometer assessment [5, 7]. Despite this, beat-to-beat monitoring was rarely used in previous studies examining this relationship [3, 5].

Furthermore, most previous studies have utilized the 1996 consensus definition for OH [3]. This definition has recently been expanded to include more sudden declines in blood pressure, as well as delayed responses [8]. The timing of a blood pressure drop may be important in managing falling risk in older adults, as it may predominantly affect different activities. For instance, a severe initial blood pressure decline may be concerning for an individual in the process of transferring. In contrast, a delayed decline could increase falling risk as a person is standing or sitting for more prolonged periods. Therefore, it is important that these alternative criteria for defining blood pressure responses be incorporated in future studies.

There is also little evidence examining how changes in cerebral blood flow velocity (CBFV) and cerebral autoregulation impact falling risk. Cerebral hypoperfusion, often preceded by a decline in upright blood pressure or OH, can lead to a fall through a syncopal episode. The loss of postural tone associated with transient loss of consciousness during syncope can mimic a falling event [3]. Only one study tested the relationship between cerebral hemodynamics and falling risk, reporting that impaired cerebrovascular reactivity to CO_2_ may increase falling risk in community dwelling older adults [9]. No studies have reported whether orthostatic changes in CBFV are associated with falling risk.

Accordingly, we aimed to: (i) test the association between blood pressure responses to orthostatic stress and falling history using different definitions for these changes in blood pressure and; (ii) test the association between cerebrovascular hemodynamic responses to orthostatic stress and falling history.

We hypothesized that individuals with more severe decreases in blood pressure at any time point during orthostatic stress would be more likely to have a positive falling history. We also hypothesized that impaired cerebral hemodynamics would be associated with a positive falling history.

## Methods

This cross-sectional study was approved by the Department Of Research Ethics at Simon Fraser University and conforms to the principles outlined in the Declaration of Helsinki. Participants were recruited from two long-term care facilities in the Greater Vancouver area and were required to understand English, be aged ≥ 65 years, and be able to follow basic instructions. Participants who had not been resident in the facilities for at least 12 months, or who were unable to ambulate, were excluded from the study. Written informed consent was obtained prior to participation from the participants or their legal designate.

### Protocols

All participants underwent a cardiovascular risk assessment, including a detailed review of their medical history using the Minimum Data Set (MDS) [10], a 12-lead electrocardiogram (ECG) recording, and a passive seated orthostatic stress test (PSOST) to evaluate cardiovascular responses to orthostatic stress.

A supine 12-lead ECG was recorded to enable the non-invasive assessment and diagnosis of electrocardiographic abnormalities (Burdick Atria 6100 12-lead ECG, Cardiac Science, Vaerloese, Denmark). This provided a qualitative screen for cardiac arrhythmia that could potentially precipitate sudden reductions in cardiac output, leading to a fall.

Participants underwent a PSOST as described previously [11]. In brief, participants remained supine for 15 min prior to being passively moved to an upright-seated position for an additional 15 min. This test has been shown to perform similarly to the head-up tilt test, considered the “gold-standard” for orthostatic stress testing [11], but without the need for prolonged standing or offsite testing away from the long-term care facilities, which was not practical with this population group. Additionally, PSOST was deemed to be more appropriate in the long-term care population in comparison to the active sit-to-stand test, another frequently used test, as the transition between supine and upright can be achieved quickly with PSOST, and does not require individuals to transition to upright under their own power, a challenging task given the mobility impairments that exist within this population group. Beat-to-beat arterial blood pressure was continuously recorded noninvasively using finger plethysmography applied to the middle finger of the right hand (Finometer® Pro and ECG Module, Finapres Medical Systems B.V., Amsterdam, The Netherlands). We applied both return-to-flow and physiocal calibrations prior to the initiation of data collection, and incorporated height correction to allow for alterations in the position of the hand relative to the heart. Heart rate and rhythm were continuously recorded using electrocardiography [lead II] (Finometer® Pro ECG Module, Finapres Medical Systems B.V., Amsterdam, The Netherlands). CBFV was measured continuously using transcranial Doppler ultrasound (Doppler-Box^™^, Compumedics Germany GmbH, Singen, Germany). Breath-by-breath end-tidal carbon dioxide (CO_2_) was determined via a nasal cannula. Data were sampled at 1 KHz using an analog:digital converter.

The MDS is a standardized assessment required or recommended for use (depending on the Province) in all long-term care facilities across Canada [10] and completed in the study facilities for all residents on a quarterly basis. We used this assessment to collect demographic information on participants (Table 1).

**Table 1:** Participant characteristics derived from the minimum data set document

Parameter	Derivation from the MDS
Cognitive function	Cognitive function was quantified by calculating a Cognitive Performance Score. This system provides a range of scores from 1 (highest level of cognitive function) to 7 (lowest cognitive function). The method has been found to have good agreement with the Folstein Mini-Mental Status Examination, considered to be the ‘gold-standard’ for detecting cognitive impairment [27]
Balance	Participant’s balance was assessed using Question G3.
Mobility	Mobility was assessed using Question G5.
Activities of daily living	These were quantified using Questions G1 and G2. A summary score was presented using the Activities of Daily Living Long [28].
Comorbidities	The presence of disease was determined from Section I1.
Medication use	This was assessed from Section O. The number of medications, as well as medication types were assessed.

The Minimum Data Set provides information about various aspects of health. This includes, but is not limited to, cognitive function, physical function, disease diagnoses, and medication use [10]. The majority of participants received this assessment on a quarterly basis. Although not a perfect instrument, it has good internal consistency, good inter-rater reliability, and high validity [27, 29, 30]. We used this assessment to gather demographic data, as well as to collect data concerning other known risk factors for falls [28, 31, 32]. These included cognitive function, mobility, impairments in activities of daily living, and medication use

Participants’ falling history was ascertained through review of fall incident report forms from both facilities. These forms are completed for every fall event reported or witnessed within each facility.

### Data analyses

Systolic (SAP), diastolic (DAP), and mean (MAP) arterial pressures were detected for each blood pressure waveform. Heart rate, stroke volume, total peripheral resistance and cardiac output were computed [11]. During the 15-min supine period, 30-s averages of all parameters were obtained to record a steady-state baseline value for each parameter.

The following variables were calculated for cardiovascular parameters: the lowest (or highest for heart rate/total peripheral resistance) 5-s upright average within the first 30 s (*Initial);* first 3 min (*Consensus);* and from 3–15 min *(Delayed).* Recovery was quantified as the final 5-s average at minutes 1, 2, 3, and 15 of upright. These intervals reflect clinically relevant time points for the varying definitions of OH [8]. We focused on SAP recovery responses because they are associated with clinical markers, including all-cause mortality [4, 12, 13].

Overall changes from baseline measurements were calculated for end-tidal CO_2_ partial pressures and CBFV measures.

We determined the number of falls that occurred during the one-year period prior to the date of the cardiovascular risk assessment. Participants were categorized as being either a faller (≥1 retrospective falls) or a non-faller (0 retrospective falls), as in previous studies [4, 7].

### Statistical analyses

Unless stated otherwise, values are reported as mean ± standard error. Level of significance was set at α = 0.05. Statistical analyses were performed using JMP 9.0.2 Statistical Software (SAS Inc. Cary, NC, United States of America). Data were tested for normality using the Shapiro-Wilk test; parametric or non-parametric tests were then used accordingly. We compared retrospective fallers and non-fallers using a Student’s *t*-test or Mann–Whitney test for continuous data, and Fisher’s Exact test for categorical variables.

## Results

### Participants

Cardiovascular risk assessments were conducted on 55 residents from two long-term care facilities. We excluded 7 residents from analysis because their retrospective falling status could not be ascertained (they had not lived in the facility for a year prior to testing). Another 2 participants did not consent to the release of medical history information, and accordingly their participant characteristics were not included. Table 2 shows participant characteristics in previous fallers and non-fallers. Higher scores for cognitive performance and activities of daily living indicate greater impairment. Fallers displayed greater impairments in activities of daily living and had a higher probability of using a mobility aid (cane, walker, or wheelchair). There were no statistically significant differences in medication use between fallers and non-fallers.

**Table 2:** Characteristics of fallers versus non-fallers

Characteristic	Retrospective fall history		*P*-value
	Faller	Non-faller	
	*n* = 26	*n* = 20	
	Mean (SD) or No (%)		
Demographics			
Age (years)	83.0 (6.8)	83.4 (9.1)	0.88
Male gender	9 (34.6)	12 (60.0)	0.14
Physical function			
Activities of daily living score	7.7 (8.3)	3.1 (5.3)	0.048
Balance			
Maintains balance unassisted	14 (53.9)	14 (70.0)	0.36
Mobility			
Unassisted	3 (11.1)	11 (55.0)	<0.005
Cane, walker or crutch	13 (50.0)	7 (35.0)	
Wheelchair	10 (38.5)	2 (10.0)	
Cognitive performance score	2.1 (1.5)	1.5 (1.1)	0.28
Medical history			
Diabetes	9 (34.6)	5 (25.0)	0.53
Atherosclerotic heart disease	5 (19.2)	0 (0)	0.058
Congestive heart failure	5 (19.2)	2 (10.0)	0.45
Arthritis	3 (11.3)	2 (10.0	1.0
Stroke	8 (30.8)	2 (10.0)	0.15
Parkinson’s disease	1 (3.9)	1 (5.0)	1.0
Alzheimer’s disease	4 (15.4)	1 (5.0)	0.37
Other dementia	7 (26.9)	9 (45.0)	0.23
Gait impairment	7 (26.9)	3 (15.0)	0.26
Medication use			
Number of medications	10.4 (4.2)	8.5 (4.2)	0.13
Antipsychotic	7 (26.9)	2 (10.0)	0.26
Antianxiety	2 (7.7)	4 (20.0)	0.38
Antidepressant	15 (57.7)	6 (30.0)	0.08
Hypnotic	7 (26.9)	2 (10.0)	0.26
Diuretic	8 (30.8)	9 (45.0)	0.37
Analgesic	17 (65.4)	11 (55.0)	0.55

All characteristics were reported as mean ± standard deviation (SD) for continuous variables, and number (%) for categorical variables. Student’s *t*-test or Mann–Whitney U tests for continuous variables, and Fisher’s Exact test for categorical variables, were used to test for differences between fallers and non-fallers

### 12-lead ECG

Although a large proportion of participants were in sinus rhythm (68.5 %), a number of individuals had atrial fibrillation (16.7 %) at the time of measurement. Additionally, 33.3 % had various forms of minor conduction abnormalities. There were no significant differences in the proportion of abnormal ECG characteristics between retrospective fallers and non-fallers.

### Cardiovascular responses to PSOST

Of those who completed the PSOST, 28 (58 %) were retrospective fallers. There were no significant differences between fallers and non-fallers during baseline for any parameter (Table 3).

**Table 3:** Baseline values for all cardiovascular parameters in fallers and non-fallers

Parameter	Fallers	Non-fallers
	Mean (SEM)	
Systolic arterial pressure (mmHg)	139.5 (4.5)	137.7 (4.5)
Diastolic arterial pressure (mmHg)	69.5 (2.2)	68.7 (2.7)
Heart rate (bpm)	75.5 (2.9)	68.8 (2.2)
Stroke volume (ml)	74.3 (8.3)	73.0 (6.8)
Cardiac output (l.min^−1^)	5.5 (0.6)	4.9 (0.4)
Total peripheral resistance (mmHg/l.min^−1^)	22.9 (2.5)	21.5 (2.4)
Systolic CBFV (cm.s^−1^)	75.5 (6.6)	73.7 (6.3)
Diastolic CBFV (cm.s^−1^)	27.6 (5.7)	24.9 (3.0)

All characteristics were reported as mean ± standard error of the mean. There were no significant differences between fallers and non-fallers for any of the variables

*Abbreviations*: *SEM* standard error of the mean, *CBFV* cerebral blood flow velocity

**Fig. 1:**
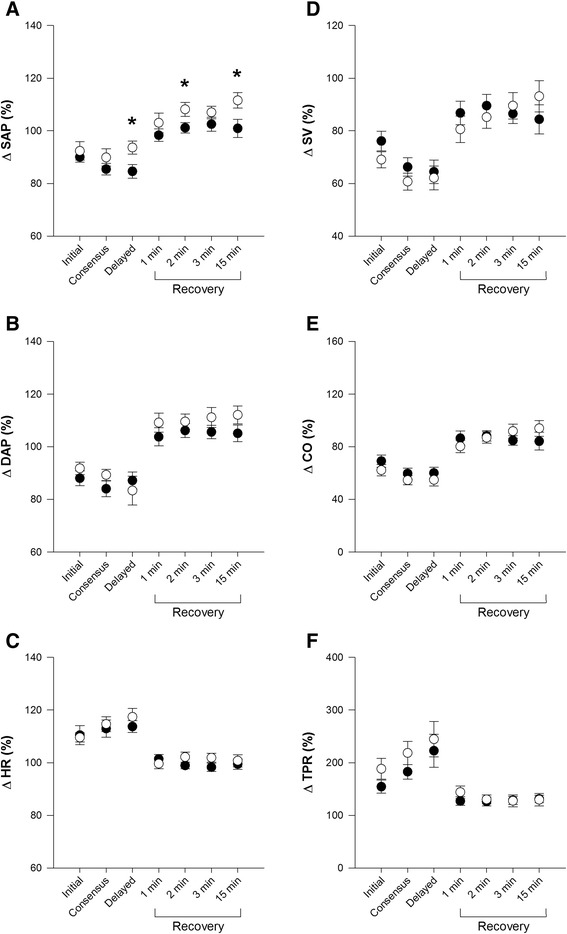
Cardiovascular Responses to the Passive Seated Orthostatic Stress Test in Fallers (Black) and Non-fallers (White). Data are shown at each time point of interest, expressed as the mean percentage change relative to supine values ± standard error of the mean. Abbreviations: systolic arterial pressure (SAP); diastolic arterial pressure (DAP); heart rate (HR); stroke volume (SV); cardiac output (CO); and total peripheral resistance (TPR). Significant differences between groups (Student’s t-test or Mann–Whitney *U* test) are denoted by * (*p* < 0.05)

During the upright phase of PSOST, fallers had a significantly larger delayed decline in SAP compared to non-fallers, as well as poorer recovery of SAP at 2 and 15 min (Fig. 1). When expressed as absolute differences, responses were qualitatively similar to the percentage changes.

**Fig. 2:**
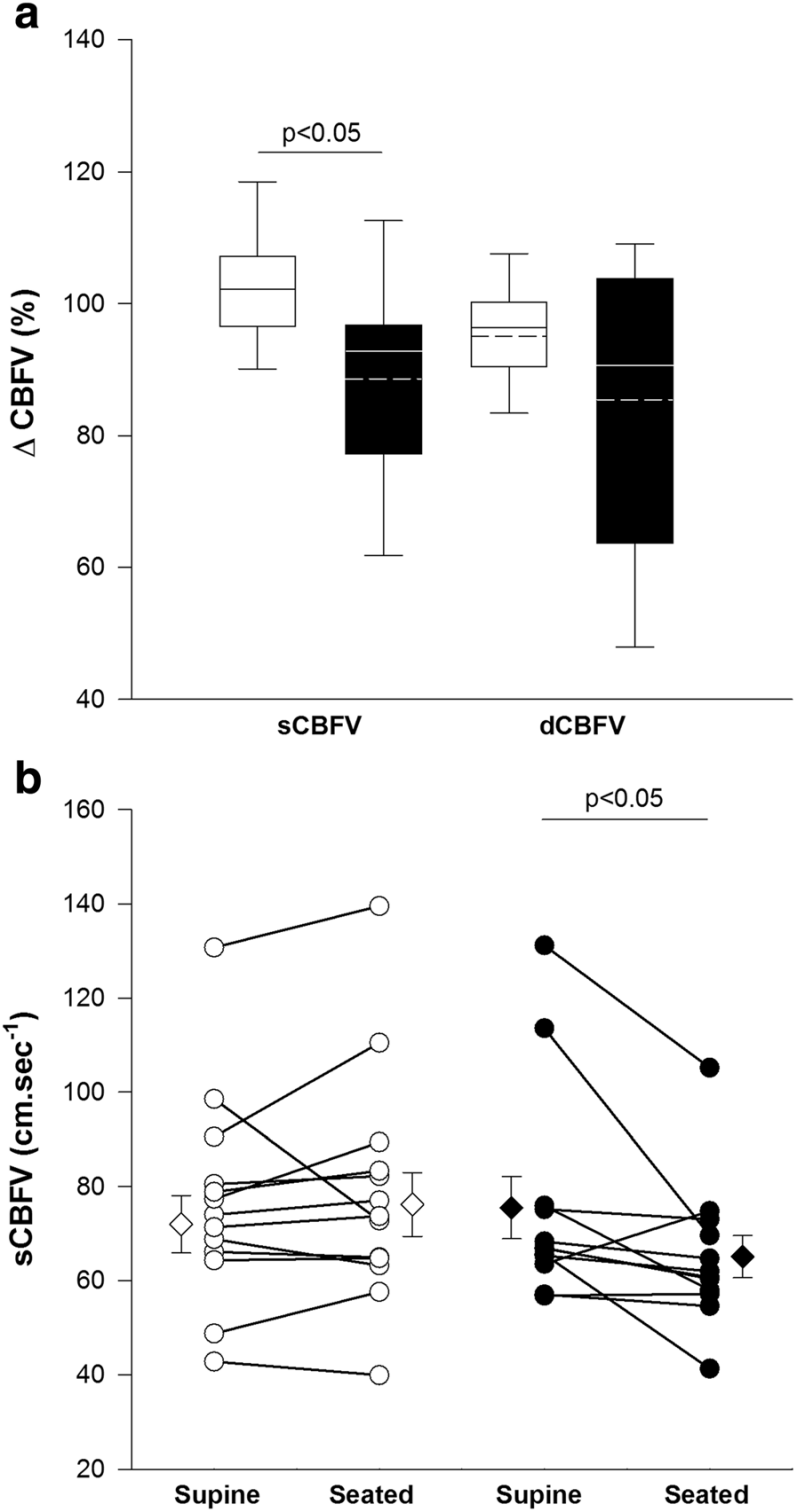
Cerebrovascular Responses to the Passive Seated Orthostatic Stress Test in Fallers (Black) and Non-fallers (White). **a**. Average seated percentage change in systolic (sCBFV) and diastolic (dCBFV) cerebral blood flow velocity in response to the passive seated orthostatic stress. The sCBFV decrease was significantly larger in fallers (-10.4 ± 4.3 cm.sec^−1^) than in non-fallers (+2.0 ± 3.0 cm.sec^−1^, *p* = 0.03). The dCBFV decrease was not significantly different between groups (fallers: −6.0 ± 3.3 cm.sec^−1^; non-fallers: −1.2 ± 1.3 cm.sec^−1^: *p* = 0.67). The solid horizontal line denotes the median; dashed horizontal line denotes the mean. **b**. Individual responses showing sCBFV in supine and seated positions in fallers (Black) and non-fallers (White). Group mean data with error bars (standard error of the mean) are shown with diamond symbols. In fallers, seated sCBFV were significantly lower than the supine values

CBFV responses were obtained in 26 (54 %) individuals (Fig. 2). Fallers had a significantly larger decline in systolic CBFV during the upright period. Changes in end tidal CO_2_ during testing did not differ between groups.

### Diagnostic criteria for OH and fall susceptibility

OH was prevalent in both groups and there were no significant differences in proportions of OH between fallers and non-fallers (Table 4)

**Table 4:** Proportion of fallers and non-fallers meeting hemodynamic criteria for orthostatic hypotension during the passive seated orthostatic stress test

Parameter	Criteria	Fallers	Non-fallers
		*n* (%)	
Initial OH	Systolic	2 (7.4)	4 (20.0)
	Diastolic	3 (11.1)	3 (15.0)
Consensus OH	Systolic	10 (37.0)	6 (31.6)
	Diastolic	9 (33.3)	7 (36.8)
Delayed OH	Systolic	11 (40.7)	5 (26.3)
	Diastolic	13 (48.2)	6 (31.6)

Initial OH was defined as a decline of > 40 mmHg systolic or > 20 mmHg diastolic within the first 30 s. Consensus OH refers to a decline of > 20 mmHg systolic or > 10 mmHg diastolic within the first 3 min. Delayed criteria were defined as declines of > 20 mmHg systolic or > 10 mmHg diastolic from 3–15 min. There were no statistically significant differences between fallers and non-fallers for any of the OH definitions. However, of the 31 participants (57.4 %) of our total sample who met hemodynamic criteria for OH at any point during the test, 16 (51.5 %) showed an OH response after 3 min time, and 8 (25.8 %) of whom exclusively had a delayed blood pressure decline

*Abbreviations*: *OH* orthostatic hypotension

## Discussion

We examined the relationships between cardiovascular responses to orthostatic stress and falling history in a small cohort of older adults living in long-term care. We showed: (i) retrospective fallers had larger delayed declines in SAP compared to non-fallers; (ii) fallers also showed poorer early (2-min) and late (15-min) recovery of SAP; (iii) fallers had a greater decline in systolic CBFV during PSOST.

The decrease in SAP during the delayed period of PSOST was larger in fallers than non-fallers. This is the first study of OH and falling to include a delayed OH definition as an explanatory variable. Most other studies used the consensus definition along with a manual sphygmomanometer [3]; this has prevented measurement of initial blood pressure declines and delayed declines, as well as potentially missing sudden perturbations that could only be detected using beat-to-beat technology. Interestingly, of the 31 participants (57.4 %) that met hemodynamic criteria for OH, 51.5 % showed an OH response after 3 min, and 25.8 % exclusively had a delayed blood pressure decline. This highlights the importance of consideration of delayed OH, and is compatible with data from elderly hospital patients in whom OH presented in 68 % of participants *after* 3 min of standing [14].

We also demonstrated that retrospective fallers had a poorer SAP recovery at 2 and 15 min of PSOST. In community dwelling older adults, Heitterachi et al. [4] demonstrated that fallers showed poorer recovery of blood pressure both immediately after tilt and at 3 min, and Pasma et al. [5] found that larger blood pressure declines within 3 min of standing were associated with impaired balance and increased self-reported falls. Neither study measured delayed OH. Interestingly, the acute recovery of SAP is also smaller in those with increasing severity of frailty [12], and this is an independent predictor for falls [15].

We found that the decline in systolic CBFV was larger in fallers than non-fallers, which may imply that cerebral autoregulation is impaired in fallers. This is supported by the marginal association reported previously between cerebral vasoreactivity to CO_2_ and prospective falling rate [9], providing further evidence that impaired autoregulation may play a role in falling risk. Impairments in CBFV and/or autoregulation may be an important mechanistic link between OH and falling risk in older adults. Reductions in CBFV (potentially exacerbated by impaired autoregulation or OH) can precipitate symptoms of orthostatic intolerance and may elicit loss of postural tone through a syncopal episode, which can present as a fall-like event [3]. Indirectly, reductions in CBFV may increase falling risk via a negative impact on cognitive functioning or balance in elderly individuals [3, 5]. Future studies must continue to explore these potential relationships.

A number of residents had underlying cardiac arrhythmias; however, there were no significant differences in the prevalence of arrhythmia between retrospective fallers and non-fallers based on the 12-lead ECG data collected. Atrial fibrillation was present in 16.7 % of participants. The prevalence of atrial fibrillation has been shown to be higher in those presenting with a non-accidental fall (absence of slipping or tripping) compared to those reporting an accidental fall [16]; we did not categorize fall types. However, arrhythmias are often transient events that may not be captured on a 12-lead ECG screen. Future studies should explore whether the presence of cardiac arrhythmia impacts falling risk in the long-term care population using longer duration arrhythmia monitoring [17].

We identified impaired systolic blood pressure responses that were related to falling; however, these findings were not reproduced using established OH definitions. Much of the emphasis in these definitions is towards how low the blood pressure falls during orthostasis, whereas our data, and that of others, suggest that blood pressure recovery may also be an important component [3]. The most recent definitions for OH now suggest that a *sustained* blood pressure decline (OH with a lack of recovery) is needed to meet criteria for consensus and delayed OH [8]. More work is needed to better understand the interactions between the decline in blood pressure during orthostasis and the subsequent recovery responses and their effect on detrimental outcomes such as falling.

We conducted our analyses in older adults who were resident in long-term care facilities. This population tends to be older, more frail, with complex pathology and more fall-prone in comparison to community-dwelling seniors [18]. Future studies should examine the links between hemodynamic and cerebrovascular responses to orthostatic stress and fall susceptibility in community-dwelling older adults. However, given the devastating impact and high incidence of falls in care facilities, we believe our data provide important mechanistic insight into the possible risk factors for falls in this vulnerable population.

### Limitations

This study has several limitations. First, we examined the theoretical relationships between hemodynamic and cerebrovascular responses to orthostatic stress and fall susceptibility; however, in older adults in long-term care, there are clearly many potential risk factors for falls. From the present study, we cannot determine the relative contribution of these hemodynamic and cerebrovascular data towards overall falling risk in these individuals.

Our data show impairments in cardiovascular and cerebrovascular control in previous fallers compared to non-fallers. Future studies should examine how these parameters relate to prospective falling risk.

Our sample size for this study was relatively small, which would obviously adversely impact the statistical power of our analyses. In addition, it was not possible to obtain measures of CBFV in every subject we tested. Insonation of the cerebral vasculature is not possible if the signal cannot penetrate the skull; this can occur due to hyperostosis, which increases in prevalence in the elderly [9]. Two large population-based studies in community–dwelling older adults reported insonation failure rates of 33 % [9] and 35 % [19]; our failure rate was comparable at 40.7 %. Our rate may be slightly higher due to differences in the population tested, as these other studies were conducted in community dwelling older adults, who were on average younger and healthier than our long-term care group.

We showed previously that the PSOST is an effective test that elicits similar acute responses in blood pressure and CBFV in comparison to tilt testing in young healthy adults [11]. This was important validation for our use of PSOST in this study, as other common orthostatic stress tests, such as tilt testing, lying-to-standing, or sit-to-stand tests were not suitable for our frail elderly population. However, we cannot be certain that the similarities between PSOST and head up tilt testing would extend to this group of older adults.

## Conclusion

Larger delayed declines in blood pressure, and impaired recovery of blood pressure in response to orthostatic stress may be risk factors for falls in older adults in long-term care. Impairments in the regulation of cerebral blood flow, the final physiological pathway implicated in fainting events, may also be an important risk factor for falling in elderly individuals. Those who were prone to falling had larger declines in systolic CBFV during orthostatic stress, providing a mechanistic link between OH, cerebral hypoperfusion and fall susceptibility. This is important given the high reported incidence of both OH and falls in older adults, and devastating impact of fall-related injury on quality of life for those affected [2, 3]. These relationships need to be further explored in prospective longitudinal studies that can better account for other falling risk factors.

Our results provide practical evidence for using the PSOST to examine blood pressure responses to orthostatic stress in long-term care facilities, particularly where individuals have mobility impairment and cannot tolerate other commonly used orthostatic stresses. One study suggested that only 67 % of residents with hypertension, and 37 % of residents with hypotension actually have this data recorded on their MDS upon entry into a long-term care facility from hospital [20]. These diagnoses are important not only for identifying the 5–10 % of individuals at risk of falls resulting directly from blood pressure impairments, but also for accurately identifying those with OH, given its association with mortality, fall susceptibility, and other severe pathologies [4, 21–25]. With proper identification, simple treatment options can be put in place to help manage these common cardiovascular disorders and reduce falling risk [26].

## Abbreviations

OH: Orthostatic hypotension
CBFV: Cerebral blood flow velocity
MDS: Minimum data set
ECG: Electrocardiogram
CO_2_: Carbon dioxide
SAP: Systolic arterial pressure
DAP: Diastolic arterial pressure
MAP: Mean arterial pressure
PSOST: Passive seated orthostatic stress test
